# Effects of clonazepam and flunitrazepam on the development cycle of *Calliphora vicina* Robineau-Desvoidy 1830 and their forensic implications

**DOI:** 10.1038/s41598-025-05766-8

**Published:** 2025-07-02

**Authors:** Lavinia Iancu, Malia Wellens, Tiberiu Sahlean

**Affiliations:** 1 Forensic Science Program, University of North Dakota, 221 Centennial Drive, Grand Forks, ND 58202 USA; 2 Forensic Science Program, University of New Haven, 300 Boston Post Rd, West Haven, CT 06516 USA; 3 Institute of Biology Bucharest, Ecology, Taxonomy and Nature Conservation Department, Romanian Academy, 296 Independentei Blvd, Bucharest, 060031 Romania

**Keywords:** Clonazepam, Flunitrazepam, * Calliphora vicina*, Development cycle, Biological techniques, Developmental biology

## Abstract

Benzodiazepines are commonly identified in drug overdose deaths worldwide. However, research on their effects on the most common necrophagous insect species is limited. In this context, the current study investigated the effects of clonazepam and flunitrazepam on the development cycle of *Calliphora vicina* Robineau-Desvoidy, 1830 (Diptera: Calliphoridae). Three blow fly colonies were reared under controlled laboratory conditions (24℃, 50% humidity, 12:12 light-dark cycle), and the experiment was carried out in triplicate. A solution of 4 mg of clonazepam and 2 mg of flunitrazepam, each dissolved in 50 mL of ultrapure water, was added to minced beef liver. The development cycle and growth rate were monitored daily, with a total of 2700 specimens weighed, covering each developmental stage except for the egg clusters. Statistical analyses using aligned rank transform (ART) ANOVA revealed significant interactions between the drug and developmental stages. Larvae exposed to benzodiazepines had higher median weights compared to controls, with more pronounced effects observed during the transition from the third instar larvae to the pupae stage. However, no differences were observed regarding the development cycle length between the three colonies. The findings suggest that clonazepam and flunitrazepam influence *C. vicina* morphology, particularly weight, which, when size is considered, has potential implications for forensic entomology in estimating the minimum postmortem interval (minPMI).

**Supplementary Information:**

The online version contains supplementary material available at 10.1038/s41598-025-05766-8.

## Introduction

Benzodiazepines are widely used to treat symptoms of anxiety and sleep disorders. Misuse is frequently reported, sometimes being associated with multi-drug overdoses, being involved in suicides, sexual assaults, and crime cases^1–3^with approximately 10,964 recorded deaths in 2022^3^. Recent data on benzodiazepines deaths^4^ showed that between 2019 and 2020 there was a 20% increase in emergency visits due to benzodiazepines overdose. This percentage included singular and multi-drug cases, the latter being a combination with opioids, like fentanyl. Overdose related deaths increased by 42.9% for the same time span, from 1,004 to 1,435 deaths^4^. The same report with data generated from 23 states showed that benzodiazepines were involved in 6,982 of the 41,496 overdose reported deaths, while opioids were identified in 6,384 of the cases. In the first half of 2020, there were 2,721 overdose deaths involving benzodiazepines, with 2,174 linked to prescription benzodiazepines and 532 to illicit benzodiazepines^4^. The most affected age group was the 25–34 years old category for overdose emergency visits, while the 45–64 years old group was the most affected category of benzodiazepine-involved deaths^4^.

In ante-mortem biological samples, benzodiazepines can be detected up to 10 days from urine^5^ 2.5 days from oral fluids^6^ 24 h from blood^7^ and up to 90 days from hair samples^8^. Once ingested, the effects of the drug will depend on the specific benzodiazepine, its concentration, the individual’s general health, and the use of other substances^9^. Moreover, the ingested substance will be metabolized primarily in the liver, breaking down into various metabolites^10^. If the ingested dose exceeds toxic levels, it can result in death of the individual^11^. Since the early 2000 s, benzodiazepines and their metabolites are often reported in postmortem specimens^12^. Compared to samples collected from living individuals, postmortem toxicological samples pose several challenges^13–15^ including the risk of decomposition, which can lead to changes in the chemical composition of the samples, and the redistribution of substances within the body after death^16,17^. Furthermore, postmortem samples may be influenced by factors such as environmental conditions and the presence of other substances that can complicate accurate analysis. Higher temperatures were found to be responsible of a faster degradation of ketazolam and chlordiazepoxide, while lorazepam and estazolam remained relatively stable when stored at −20 °C and − 80 °C^15^. Moreover, the biotransformation of parent drugs to metabolites can continue during the storage of the postmortem samples^14^. After death, during the fresh stage of decomposition or upon body discovery, biological tissues and fluids will be sampled for toxicological analyses during the autopsy^18^. If a body is found in an advanced stage of decomposition, identifying benzodiazepines from decomposed samples can be challenging. In such cases, when present, necrophagous insect species collected from the body can serve as an alternative sample type for drug detection^1^.

The development of forensic entomotoxicology expanded decades ago^19^ focusing primarily on the detection of drugs from different insect species^20–22^. Necrophagous insect species, mainly calliphorids, were used for these investigations, as these are typically the first insects to arrive and colonize a body minutes to hours after death^23,24^. Their presence and activity is influenced by a number of factors, starting with the environmental temperature, geographical location, manner of death, up to the presence of different drugs in the feeding substrate, represented by decomposed organic matter^24^. Different analytical methods, such as liquid chromatography-tandem mass spectrometry (LC-MS-MS) were tested to detect benzodiazepines from different Calliphoridae species, including *Calliphora vicina* Robineau-Desvoidy, 1830^1^. Other studies^25^ focused on developing non-destructive methods for flunitrazepam identification from various developmental stages of *Chrysomya megacephala* (Fabricius, 1794) (Diptera: Calliphoridae), or used Fourier-transformed infrared (FTIR) microscopy^26^ as well as other identification procedures to identify benzodiazepines from insect tissues^27–31^. Several benzodiazepines could be detected from insect samples, including flunitrazepam, clonazepam^1^ diazepam^22^ and nordiazepam^32^.

The necrophagous insect species developmental stages, and arthropod community succession are analyzed for the estimation of the minimum postmortem interval (minPMI)^24^. Since this estimation is often based on the developmental stages, and knowing that the rate of development can be influenced by different factors including various drugs and toxins, several studies focused on understanding the influence of different drugs on the growth rate of various Calliphoridae species^33–35^. To better understand the growth rates as they relate to time, several studies^36–39^ have investigated the development of calliphorids, including *C. vicina*. This species is oviparous and can lay up to 300 eggs in a cluster, primarily in body orifices (e.g. mouth, nose) and open wounds^23^. The larvae hatch approximately 24 h later, feeding and growing for several days through the first, second, and third instars^40^. Towards the end of the third larvae stage, the specimens cease feeding and migrate away from the food source (e.g. animal carcass, human body) to pupate. During the pupal stage, which lasts for several days, the larval tissues undergo metamorphosis, culminating in the emergence of teneral specimens (newly hatched adults)^23^. Knowing that the life cycle dynamics is species-specific and primarily temperature dependent, these studies monitored the growth rates at different temperatures to better understand the developmental stages’ temperature dependency, including changes in larvae length and survival rates^36,41^. This approach aims to achieve more accurate reference data for the minPMI estimation, recognizing that temperature-dependent growth rates vary significantly among different insect species. Reference rearing data is commonly used in forensic cases involving insect evidence^42^ taking into account the macro and micro environment of each crime scene, including the presence of drugs in the decomposed tissues.

Overall, research focused on the effects of benzodiazepines on blowfly species development are scarce. In this context the current study aimed to answer the following questions: (1) Are flunitrazepam and clonazepam causing changes in the growth rate of *C. vicina*, a primary Calliphoridae colonizer? (2) Are there any differences in the overall development cycle times between the three treatments (control, clonazepam, and flunitrazepam)?

## Results

### Benzodiazepines influence on *C. vicina* development cycle

**Fig. 1:**
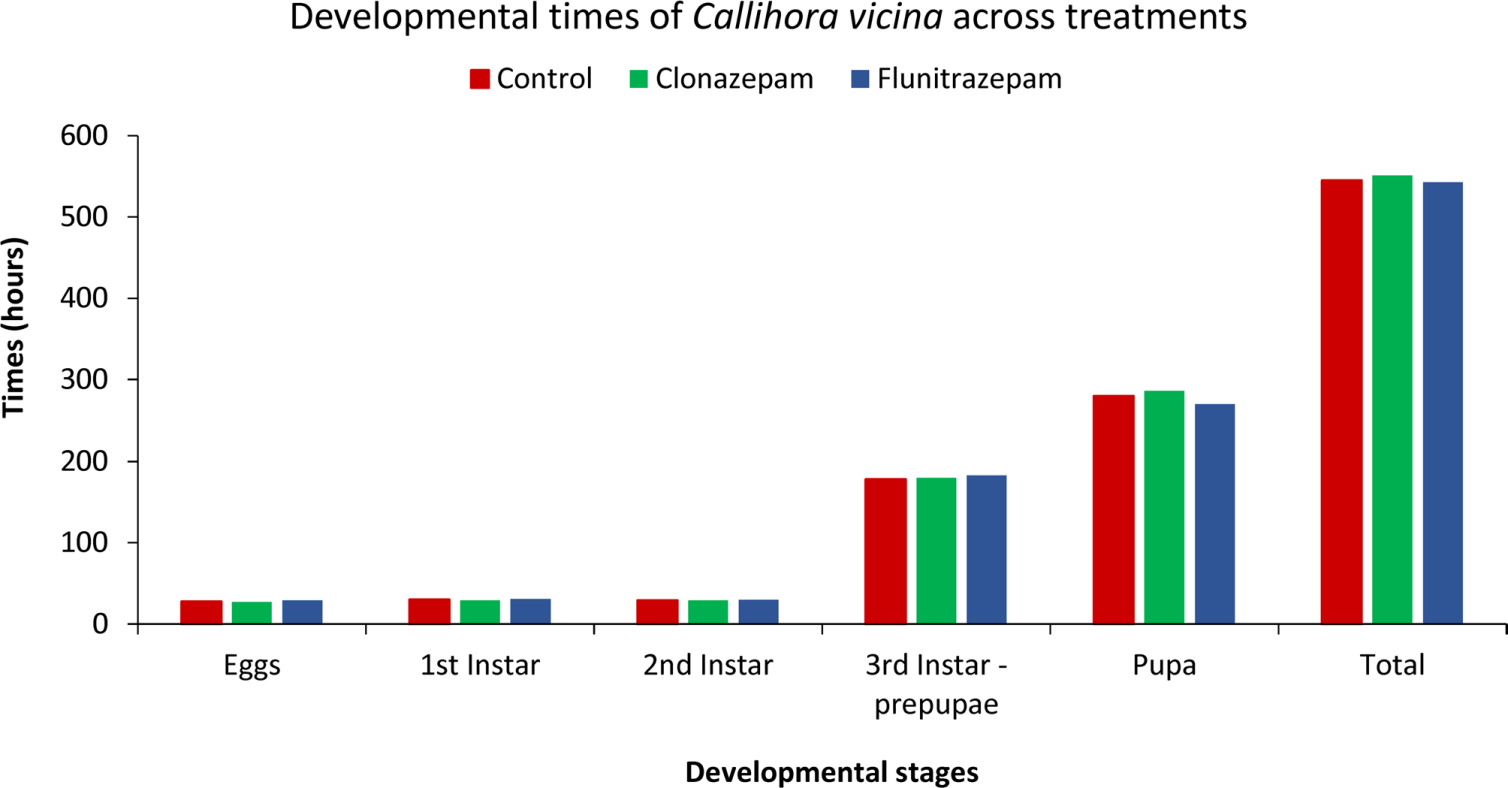
Developmental times (in hours) for *Calliphora vicina* across different treatments (control, clonazepam, and flunitrazepam).

There were no notable differences observed in the time length of the development cycle between the two colonies fed with inoculated liver and the control colony (Fig. 1, Table S1). All specimens took approximately 546 h to reach the teneral stage from the moment of oviposition (Table S1). The time differences in Table 1 account for the variations in weighing times for each colony.

**Table 1:** Developmental times per treatment and measurement schedule.

Treatment	Developmental stage	Time at weighing (hours)
Control	First Instar	26
Control	Second instar	26
Control	Third instar	125
Control	Pupae	266
Control	Teneral	284
Clonazepam	First Instar	27.5
Clonazepam	Second instar	27.5
Clonazepam	Third instar	126.5
Clonazepam	Pupae	267.5
Clonazepam	Teneral	290
Flunitrazepam	First Instar	29
Flunitrazepam	Second instar	29
Flunitrazepam	Third instar	128
Flunitrazepam	Pupae	269
Flunitrazepam	Teneral	276

### Benzodiazepines effect on C. vicina weight

**Fig. 2:**
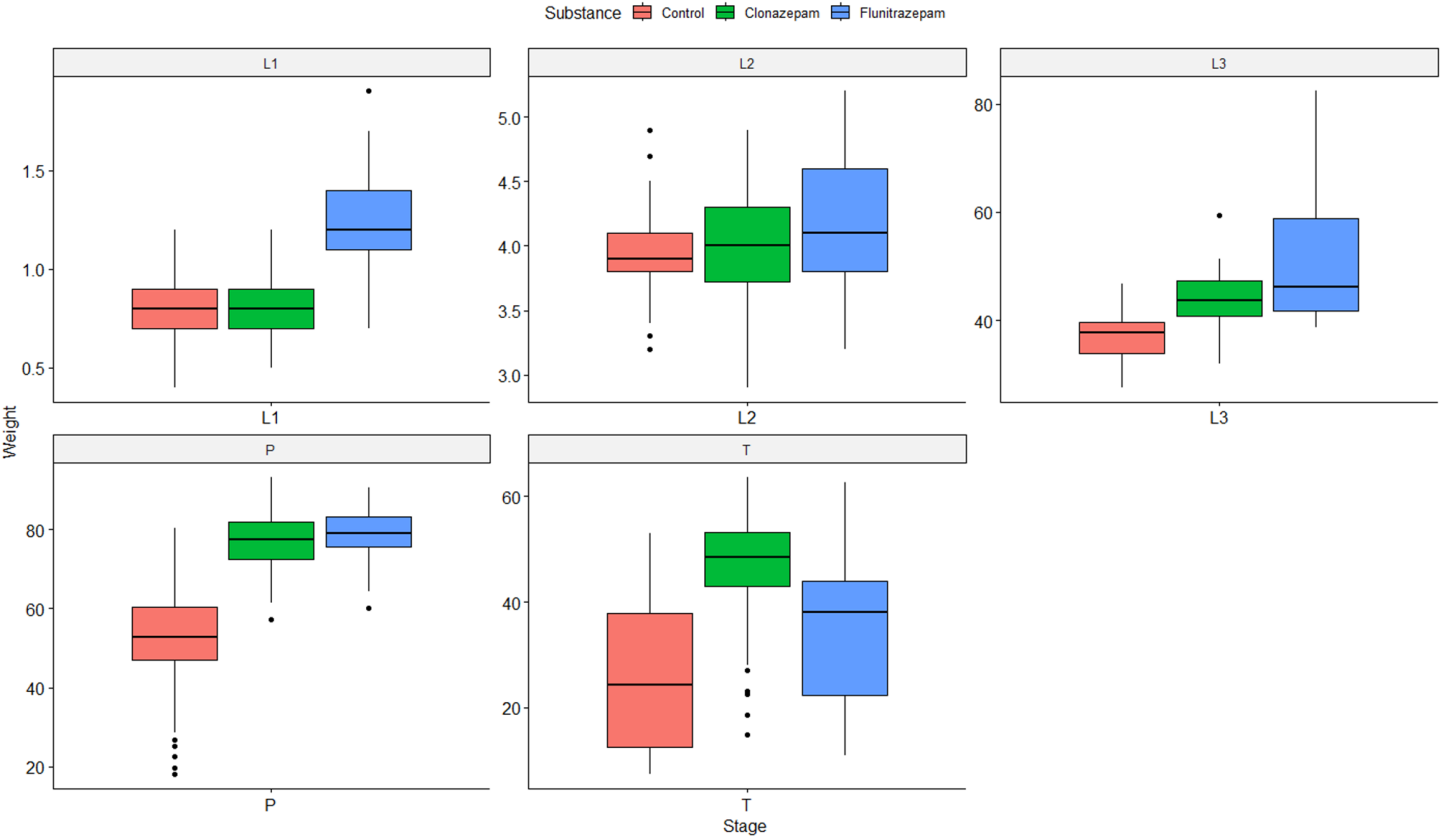
Boxplots illustrate the weight response (in mg) of *Calliphora vicina* at various developmental stages (L1 = first instar larvae; L2 = second instar larvae; L3 = third instar larvae; P = pupae; T = teneral) under different exposure conditions: control (red); clonazepam (green); flunitrazepam (blue).

**Fig. 3:**
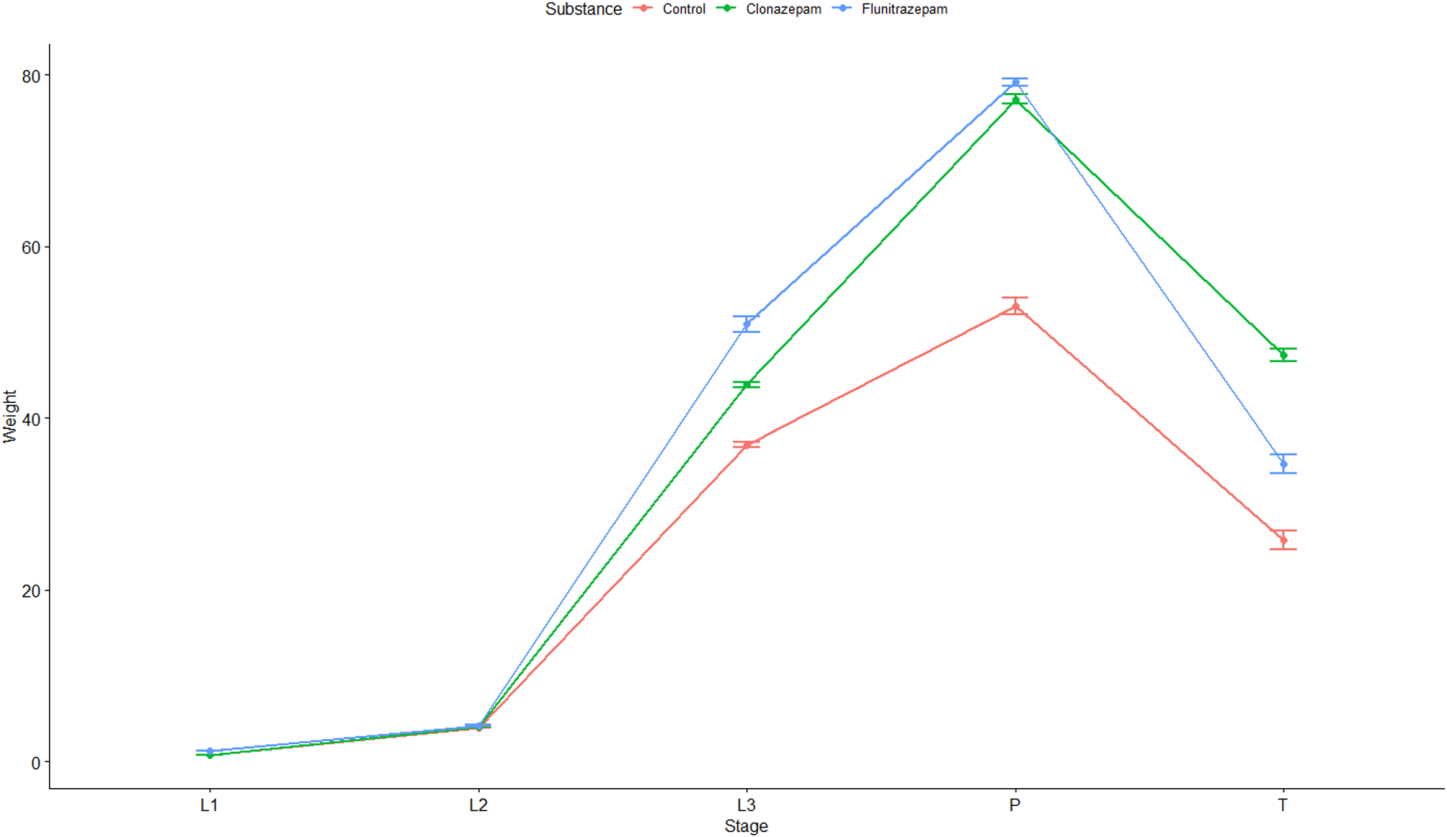
Interaction plot showing the mean weight (in mg) of *Calliphora vicina* at various developmental stages (L1 = first instar larvae; L2 = second instar larvae; L3 = third instar larvae; P = pupae; T = teneral) under different feeding conditions: control (red); clonazepam (green); flunitrazepam (blue). The mean weight of the third instar larvae and pupae is higher in the benzodiazepine-exposed groups compared to the control group.

The ART ANOVA test revealed significant interactions between the substance used on the feeding substrate and developmental stage, related to the weight of the larvae (F (8, 28.628) = 8.2309, *p* < 0.0001). The median weight of the larvae was significantly higher in the colonies exposed to benzodiazepines compared to those with no treatment (clonazepam: *t* = −6.164, *df* = 28.1, *p* < 0.0001, flunitrazepam: *t* = −5.018, *df* = 28.1, *p* = 0.0001). However, there was no significant difference in the median weight of larvae from colonies where flunitrazepam and clonazepam were present (*t* = 1.278, *df* = 28.4, *p* = 0.4191). The pairwise test of differences in pairwise combinations of levels between factors in interactions did not reveal significant differences in median weight from control colonies compared to colonies exposed to benzodiazepines when larvae developed from the first instar larvae (L1) to the second instar larvae (L2), but differences appeared later in development, as the median weight was significantly different between control and benzodiazepine specimens moving from the third instar larvae (L3) to the pupae stage (Table S1, Fig. 2). Overall, differences were more pronounced for flunitrazepam, as the median weight was significantly different for the larvae exposed to this substance compared to control in almost all developmental stages, except for the first and second instar larvae (Fig. 2). The differences were statistically significant for clonazepam compared to control only when transitioning from the third instar larvae to the pupae stage (Table S2, Fig. 2). Although the median weight was higher for the larvae grown on flunitrazepam inoculated liver, differences between the two benzodiazepines were only significant moving from pupae to teneral stage (Table S1, Fig. 3).

There was no evidence of association between the feeding substrates and the frequency of hatched males and females (χ^2^ = 0.12585, df = 2, *p* = 0.939).

## Discussion

Whether used therapeutically or obtained illegally, benzodiazepines can negatively impact the overall health of an individual, potentially leading to life-threatening conditions and, in some cases, death^4,43^. The pharmacokinetics and postmortem distribution of each drug in this class vary and understanding how these factors affect different necrophagous insect species could provide valuable reference data for when estimating the minPMI.

Differences in the weight of fly specimens collected from decomposed remains can influence the estimates of the minPMI, as heavier larvae typically indicate more advanced development^44^. In the current experiment, both benzodiazepines influenced the weight of the specimens exposed to the feeding environment spiked with these drugs. This response may be due to the nervous system malfunction. In the early 1987^45^ researchers used nerve cells from the thoracic ganglia of two locust species to test the Gamma-aminobutyric acid (GABA), the main inhibitory neurotransmitter in the insect nervous system, and muscimol, to examine how they respond to specific voltages. They found that, as a reaction, the cells become more negatively charged, while the presence of flunitrazepam enhanced the rapid response without extending the duration of the cellular reaction. As demonstrated^45,46^ insect GABA receptors have sites that can be modulated by benzodiazepines, such as flunitrazepam and clonazepam.

Benzodiazepines can enhance the effects of GABA, increasing the opening of chloride channels, leading to a grater inhibition of the neuronal activity^47^. This, in turn, decreases signal transmission between neurons, modulating behavior and physiology. Nevertheless, in this earlier study^45^ flunitrazepam did not appear to affect the resting state of the cells or their basic electrical properties. The results suggested that the flunitrazepam effect is not due to blocking GABA uptake but rather to its direct action on the GABA receptors. The overall activity of the neurons is reduced, leading to a potential calming effect^48^. Although GABA is widespread among vertebrate and invertebrate species^49^ previous studies have shown that the benzodiazepines binding sites for insect GABA receptors are pharmacologically different than in vertebrate species^50^. A study on *Periplaneta americana* (Linnaeus, 1758) (Coleoptera: Blattidae)^46^ confirmed once again that there is a clear pharmacological difference between the insect and mammalian native GABA-gated chloride channels when considering the benzodiazepines effects. These neuronal modifications could explain the changes in body mass, as it has been demonstrated that flunitrazepam can bind to the insect neuronal tissue^51^.

The cause of larvae and pupae weight gain could be explained by the disruption of normal neuronal activities. A previous study^52^ investigating the associated effect of ethanol and flunitrazepam showed that only the 7-aminoflunitrazepam impacted the growth rate, causing longer lengths of the larvae stages. Another study^53^ focused on the effect of flunitrazepam on *C. megacephala* species growth rates revealed that the drug impacted the weight gain of the pupae and adults. These findings are supported by the current results, especially for the pupa stage, where the median weight of the specimens exposed to benzodiazepines was significantly higher than the control specimens. Noteworthy, there were no significant differences between the two drugs regarding the growth rate. Moreover, Baia and collaborators^53^ used different flunitrazepam concentrations and found that the pupae were heavier when using a higher concentration in the feeding environment. The development cycle of several calliphorid species has been affected by the presence of carbamazepine and clobazam in the feeding substrate^54^. Clonazepam studies are scarce, with only a few investigating the effects on a sarcophagid species^55^ and a phorid species^56^. Clonazepam was found to have an impact on the increase in weight of the third larval stage^55^ and on the acceleration of the development cycle^56^.

Carvalho and collaborators^22^ found that diazepam can have an influence on the minPMI estimation when present in the feeding substrate of *Chrysomya albiceps* (Wiedemann, 1819) and *Chrysomya putoria* (Wiedemann, 1830), as it alters the growth of these two calliphorid species. Other substances, such as cocaine^57^ methamphetamine^58^ and ketamine^33^ can accelerate the development cycle of necrophagous insects. Results should be interpreted with caution, as the same substance, such as ketamine, can either accelerate the development^33^ or have no correlation^59^ when tested on two different calliphorid species. The presence of different toxins can have a reverse effect as well, causing delays of the development cycle^60,61^. When tested on *C. megacephala*, chemotherapeutic drugs were found to influence the survival rates and sex ratios^62^. A recent study^63^ investigated the effect of antibiotics, like ceftriaxone and levofloxacin, on *Calliphora vomitoria* (Linnaeus, 1758) growth cycle and determined that the larval development and pupation were influenced in different ways.

The length of the development cycle remained unaffected, providing similar findings as previously reported^53^. Nevertheless, each drug can have a different impact on the life cycle length of various necrophagous insect species^64,65^. In the current experiment the development cycle had a mean duration of 546 h for all three colonies. When comparing development times for the same species, temperature and relative humidity, even in a controlled insect rearing chamber, must be considered when analyzing the development cycle length.

When drugs are present in the feeding substrate at the concentrations reported in this study and considering that they did not influence the duration of the development cycle, it can be concluded that their impact on the minPMI estimation could be minimal. However, caution should be exercised when analyzing different life stages regarding weight and length changes, as these drugs affected the weight rates in later developmental stages, which can introduce errors in the results interpretation.

## Materials and methods

### Rearing conditions

The experiment received approval from the Institutional Biosafety Committee, University of North Dakota (IBC-202212-006), complying with the safety and standards protocols.

**Fig. 4:**
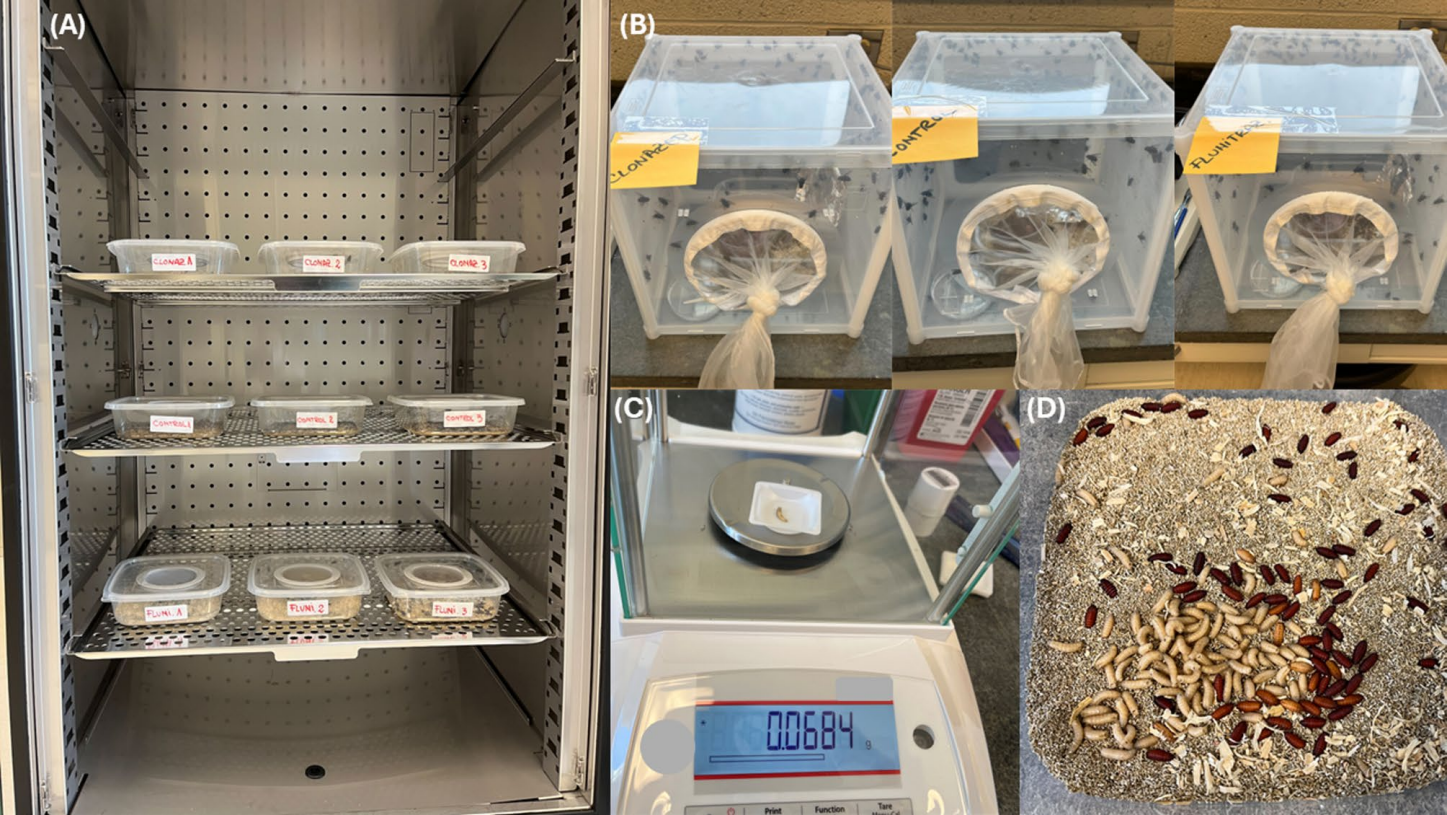
Experimental setup: (**A**) Rearing chamber; (**B**) Rearing cages; (**C**) Weighing process; (**D**) Pupariation.

*C. vicina* third instar larvae were acquired from a local live bait shop. Following emergence, three colonies of 300 *C. vicina* adults each were reared under constant laboratory conditions (24℃, 50% humidity, 12:12 light-dark cycle) using an insect rearing chamber (Caron, USA) (Fig. 4A). The adults were provided daily with water and honey, while 200 g of minced beef liver was placed in each rearing cage until oviposition occurred five days later (Fig. 4B). Several larvae and adult specimens were taxonomically identified under a Leica S9 D stereomicroscope (Leica, USA) for species confirmation, according to the taxonomic identification keys for the blowflies of North America^40^.

The concentrations of both drugs were based on the toxic dose per kilogram of body weight^66,67^. Specifically, 4 mg of clonazepam and 2 mg of flunitrazepam were each mixed with 50 mL of ultrapure water using a magnetic stirrer (Thermo Scientific, USA), resulting in concentrations of 0.08 mg/mL for clonazepam, and 0.04 mg/mL for flunitrazepam. The concentrations were not further diluted beyond the initial preparation. The solutions were then spiked into the minced beef liver and manually homogenized, ensuring that no excess liquid escaped from the feeding substrate container.

After oviposition, the egg clusters were transferred to nine rearing containers: three containing minced livers spiked with clonazepam, three containing minced livers spiked with flunitrazepam, and three rearing containers with only minced liver used as control. Each rearing container included 200 g of minced beef. Three egg clusters, each counting approximately 250 eggs, were transferred to each container. The transfer of the egg clusters was randomized to ensure that the same number of clusters were placed in each container. All nine containers were maintained under the same rearing conditions as the adult colonies.

Development cycle monitoring took place daily (every 6 h). Fifty insect specimens, representing adults, first instar larvae, second instar larvae, third instar larvae (mid-stage), pupae, and teneral stages, were weight from each rearing container, totaling 2700 measurements, using an analytical balance (Ohaus Pioneer, USA). During each measurement, the specimens were randomly selected and placed in a second container, until the end of the weighing process, to avoid measuring the same specimen twice (Fig. 2C). The non-destructive approach was used to allow accounting for pupariation and mortality rates, and final adults emergence rates (Fig. 2D). Weighing 450 specimens across all containers for each developmental stage took approximately 4 h. This information was included in the developmental timetable to account for the time differences between measurements. Specimens were monitored daily for developmental stage confirmation, based on the morphological features specific to each stage, especially the larvae stages that were accounted for, based on the number of posterior respiratory slits (spiracles).

### Statistical analysis

Statistical analyses were conducted using the R software environment^68^. Aligned Rank Transform (ART) ANOVA, implemented in the package ARTool^69,70^, was used to analyze the effect of benzodiazepines on the weight of *C. vicina* specimens through the developmental stages (first instar larvae, second instar larvae, third instar larvae, pupae, and teneral); an unique ID composed of the developmental stage and container (e.g. L1 CT1 – stage 1 control container 1) was used as a random factor to account for the potential variability between the three replicates. Pairwise tests to evaluate the influence of each drug at different developmental stages were also conducted using the *art.con* function^71^ from the same package. The independence of the resulting sex of individuals from the colonies exposed to the drugs was analyzed using the χ^2^ test of independence. All analyses were performed at a confidence level of 95%. Plots were produced using the package ggpubr^72^.

## Electronic supplementary material

Below is the link to the electronic supplementary material.

Supplementary Material 1

## Data Availability

All data presented in this study are included within the current manuscript and the supplemental material.
