# Supplementary material for: Effects of clonazepam and flunitrazepam on the development cycle of Calliphora vicina Robineau-Desvoidy 1830 and their forensic implications
Source: Sci Rep. 2025 Jul 2;15:22773. doi: 10.1038/s41598-025-05766-8 (PMC12219213; doi:10.1038/s41598-025-05766-8)
Supplement: Supplementary file 1 — Supplementary Material 1 [file 41598_2025_5766_MOESM1_ESM.docx]

**Table S1. Developmental data for *Calliphora vicina* colonies (control, clonazepam, and flunitrazepam), reared at 24℃.**

| *Calliphoria vicina* | | | |
| --- | --- | --- | --- |
| Constant temperature rearing 24°C | | | |
|  | Control | Clonazepam | Flunitrazepam |
| Stages | Time (hours) | Time (hours) | Time (hours) |
|  |  |  |  |
| Eggs | 28 | 27 | 29 |
| 1st Instar | 30 | 29 | 31 |
| 2nd Instar | 29 | 29 | 30 |
| 3rd Instar - prepupae | 178 | 180 | 183 |
| Pupa | 280 | 286 | 270 |
| Total | 545 | 551 | 543 |

**Table S2. Test of differences in pairwise combinations of levels between factors in interactions.**

|  | **Substance pairwise** | **Stage pairwise** | **t.ratio** | **df** | **p.value** |
| --- | --- | --- | --- | --- | --- |
| 1 | Control - Clonazepam | L1 - L2 | 0.109764 | 28.04934 | 0.913378 |
| 2 | Control - Flunitrazepam | L1 - L2 | -0.181 | 28.04934 | 0.857669 |
| 3 | Clonazepam - Flunitrazepam | L1 - L2 | -0.29076 | 28.04934 | 0.773371 |
| 4 | Control - Clonazepam | L1 - L3 | 1.878027 | 28.04934 | 0.070810 |
| 5 | Control - Flunitrazepam | L1 - L3 | 2.225065 | 28.04934 | 0.034294 |
| 6 | Clonazepam - Flunitrazepam | L1 - L3 | 0.484947 | 29.42438 | 0.631305 |
| 7 | Control - Clonazepam | L1 - P | 4.973842 | 28.04934 | 0.000030 |
| 8 | Control - Flunitrazepam | L1 - P | 4.583999 | 28.04934 | 0.000086 |
| 9 | Clonazepam – Flunitrazepam | L1 - P | -0.38984 | 28.04934 | 0.699597 |
| 10 | Control - Clonazepam | L1 - T | 4.633435 | 28.04934 | 0.000075 |
| 11 | Control - Flunitrazepam | L1 - T | 2.001768 | 28.04934 | 0.055066 |
| 12 | Clonazepam - Flunitrazepam | L1 - T | -2.63167 | 28.04934 | 0.013654 |
| 13 | Control – Clonazepam | L2 - L3 | 1.768263 | 28.04934 | 0.087894 |
| 14 | Control - Flunitrazepam | L2 - L3 | 2.406065 | 28.04934 | 0.022960 |
| 15 | Clonazepam - Flunitrazepam | L2 - L3 | 0.891259 | 29.42438 | 0.380021 |
| 16 | Control - Clonazepam | L2 - P | 4.864078 | 28.04934 | 0.000040 |
| 17 | Control - Flunitrazepam | L2 - P | 4.765 | 28.04934 | 0.000053 |
| 18 | Clonazepam - Flunitrazepam | L2 - P | -0.09908 | 28.04934 | 0.921781 |
| 19 | Control - Clonazepam | L2 - T | 4.523671 | 28.04934 | 0.000102 |
| 20 | Control - Flunitrazepam | L2 - T | 2.182768 | 28.04934 | 0.037583 |
| 21 | Clonazepam - Flunitrazepam | L2 - T | -2.3409 | 28.04934 | 0.026573 |
| 22 | Control - Clonazepam | L3 - P | 3.095815 | 28.04934 | 0.004421 |
| 23 | Control - Flunitrazepam | L3 - P | 2.358935 | 28.04934 | 0.025525 |
| 24 | Clonazepam - Flunitrazepam | L3 - P | -1.02971 | 29.42438 | 0.311536 |
| 25 | Control - Clonazepam | L3 - T | 2.755408 | 28.04934 | 0.010181 |
| 26 | Control - Flunitrazepam | L3 - T | -0.2233 | 28.04934 | 0.824923 |
| 27 | Clonazepam - Flunitrazepam | L3 - T | -4.16242 | 29.42438 | 0.000251 |
| 28 | Control - Clonazepam | P - T | -0.34041 | 28.04934 | 0.736086 |
| 29 | Control - Flunitrazepam | P - T | -2.58223 | 28.04934 | 0.015328 |
| 30 | Clonazepam - Flunitrazepam | P - T | -2.24182 | 28.04934 | 0.033064 |
